# Dissecting the p53-Mdm2 feedback loop *in vivo*: uncoupling the role in p53 stability and activity

**DOI:** 10.18632/oncotarget.1797

**Published:** 2014-03-14

**Authors:** Vinod Pant, Guillermina Lozano

**Affiliations:** ^1^ Department of Genetics, M.D. Anderson Cancer Center, Houston, Texas

**Keywords:** Autoregulation, MEFs, Mdm4, mouse model, E3-ligase, p53 degradation, P2 promoter

## Abstract

The p53-Mdm2 feedback loop is thought to be the main mechanism by which p53 autoregulates its levels and activity after DNA damage. We tested this paradigm in a genetically engineered mouse model in which the feedback loop was disrupted by point mutations in the p53 binding site of the Mdm2 promoter. We noted that while the p53-Mdm2 feedback loop is required to regulate p53 activity especially in the hematopoietic system in response to DNA damage, its role in development and in regulating the stability of p53 is dispensable. In the present study we have extended our characterization of this mouse model and show that the kinetics of p53 degradation is also unchanged in mouse embryonic fibroblasts (MEFs). Additionally, MG132 experiments indicate that other E3-ligases regulate p53 stability. Also, Mdm4 cooperates in inhibition of p53 activity and levels in these mice. Finally, we show in this system that enhanced acute p53 response does not promote aging or protect against late term tumorigenesis. We also discuss future perspectives for this study.

## INTRODUCTION

The “Guardian of the genome” and tumor suppressor, p53 induces cell cycle arrest, senescence or apoptosis in cells that have experienced DNA damage following genotoxic insults [1]. This genoprotective function of p53 necessitates its level and activity be tightly regulated in cells under both homeostatic and post-DNA damage conditions. A range of p53 regulators have been identified for this function [2]. However, elegant genetic studies thus far have confirmed only Mdm2 and Mdm4 as the major regulators of p53 [3-5]. These two homologous proteins directly bind p53 through their N-terminal domains and inhibit p53 transcriptional activity. In addition, Mdm2 encodes an E3 ubiquitin ligase for p53 and promotes its degradation via the 26S proteosome machinery [6-8]. Interestingly, Mdm2 in itself is a transcriptional target of p53. The P2-promoter of *Mdm2* carries two distinct p53 response elements wherein stress induced p53 binds and promotes transcription of *Mdm2* from this alternative promoter [9, 10]. This dynamic relationship between the two proteins thus results in a feedback loop in which DNA damage-activated p53 promotes *Mdm2* transcription while the translated Mdm2 protein inhibits p53 functions. Since its discovery in the early 90's, the feedback loop is considered the major pathway necessary for regulating the post-stress levels and activity of p53 in a cell [6, 11-13]. Evidence in support of this idea is derived mostly from correlative studies which present an inverse correlation between Mdm2 abundance and levels/activity of the p53 protein. We recently generated a mouse model to address the importance of the feedback loop and reported that while the feedback loop is required to regulate p53 activity, especially in the hematopoietic system after DNA damage, its role during development and in regulating p53 stability is dispensable [14]. However, questions remained in terms of the role of the feedback loop in regulating p53 half-life and the role of Mdm4 in Mdm2-mediated p53 degradation. Here we have addressed these questions by further characterization of cells and tissues from these mice. We show that in the absence of DNA damage p53 half-life remains similar between *Mdm2^+/+^* and *Mdm2^P2/P2^* MEF cells. However, after DNA damage p53 degradation is delayed in *Mdm2^P2/P2^* cells. In addition, Mdm4 contributes towards inhibition of p53 activity and stability.

## RESULTS

### *Mdm2^P2/P2^* mice exhibit no phenotypic abnormalities

We introduced point mutations in the two p53 binding sites at the *Mdm2* P2 promoter [14]. These targeted alterations were specifically designed to inhibit binding of stress induced p53 to the *Mdm2* promoter and thus abrogate the p53-Mdm2 feedback loop. Analysis of the mutant *Mdm2* P2 promoter sequence against the TRANSFAC database (www.gene-regulation.com) confirmed that no inadvertent novel transcription factor binding sites were created. A targeting vector with the mutant *Mdm2* P2-promoter was used for generating the *Mdm2^P2/P2^* knock-in allele. To our surprise, even in the absence of p53 autoregulation, homozygous *Mdm2^P2/P2^* mice were born in normal Mendelian ratios and developed normally [14]. In contrast, previous studies have shown that mice which completely lack *Mdm2* are embryo lethal and die due to increased p53 activity [3, 4]. In addition, mice with reduced Mdm2 expression exhibit increased p53 activity which manifests many different phenotypic alterations such as hyperpigmentation of skin, kinky tail, small size, lymphopenia etc [15, 16]. However, *Mdm2^P2/P2^* mice did not exhibit any such phenotypic anomalies and survived till adulthood suggesting normal levels of p53 activity (data not shown). While p53 activation is well documented during embryonic development [17, 18], our studies indicate that the feedback loop is dispensable and that basal Mdm2 levels (from P1 promoter) are sufficient to regulate p53 activity.

### p53 stabilization in response to stress stimuli in feedback loop deficient MEFs

**Figure 1:**
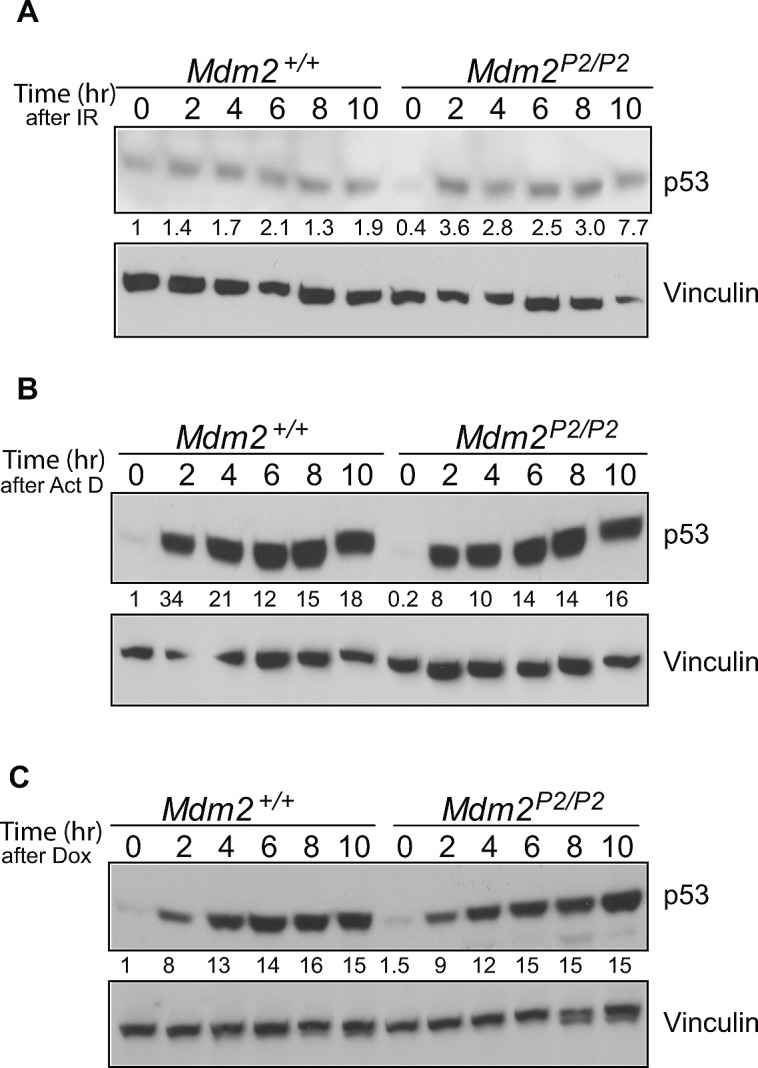
p53 is induced in response to stress signals similarly in *Mdm2^+/+^* and *Mdm2^P2/P2^* MEFs (A) Western blot analysis for p53 stability in 6 Gy IR irradiated *Mdm2^+/+^* and *Mdm2^P2/P2^* MEFs at different time points. (B) Western blot analysis for p53 stability in Actinomycin D treated *Mdm2^+/+^* and *Mdm2^P2/P2^* MEFs at different time points. (C) Western blot analysis for p53 stability in Doxorubicin treated *Mdm2^+/+^* and *Mdm2^P2/P2^* MEFs at different time points. Numbers at the bottom denote p53 fold induction normalized to vinculin controls and relative to untreated *Mdm2^+/+^* controls.

We had previously examined p53 stabilization in *Mdm2^+/+^* and *Mdm2^P2/P2^* mouse tissues after exposure to sublethal doses of ionizing radiation (IR) [14]. Similar levels of p53 stabilization were observed in various tissues of both genotypes, although the levels were slightly higher in *Mdm2^P2/P2^* mice. Here we extended this evaluation to early passage mouse embryonic fibroblasts (MEFs) from *Mdm2^+/+^* and *Mdm2^P2/P2^* mice in response to other types of stress stimuli which have been described for p53 stabilization. First, we irradiated *Mdm2^+/+^* and *Mdm2^P2/P2^* MEFs with 6 Gy IR and harvested them at different time points to assess p53 stability (Figure 1A). p53 was similarly stabilized in both genotypes and remained stable for the 10 hour duration of the experiment. Next, we tested p53 stability after exposure to Actinomycin D, an inhibitor that mimics ribosomal stress conditions (Figure 1B) [19]. Again, similar levels and duration of p53 stabilization were observed in MEFs of *Mdm2^+/+^* and *Mdm2^P2/P2^* genotypes. Finally, we tested doxorubicin as an inducer of p53 stability in these MEFs (Figure 1C). Genotoxic stress created by Doxorubicin exposure also stabilized p53 similarly in both genotypes. Absence of Mdm2 leads to spontaneous stabilization of mutant and wild-type p53 [16, 20]. However, we did not observe p53 stabilization in untreated cells in these experiments. This indicates that absence of the feedback loop in itself does not constitute a stress signal to induce wild-type p53 stabilization. Overall these experiments indicated that p53 stabilization in response to various stress signals is intact in feedback deficient MEFs.

### Role of p53-dependent Mdm2 in p53 degradation

**Figure 2:**
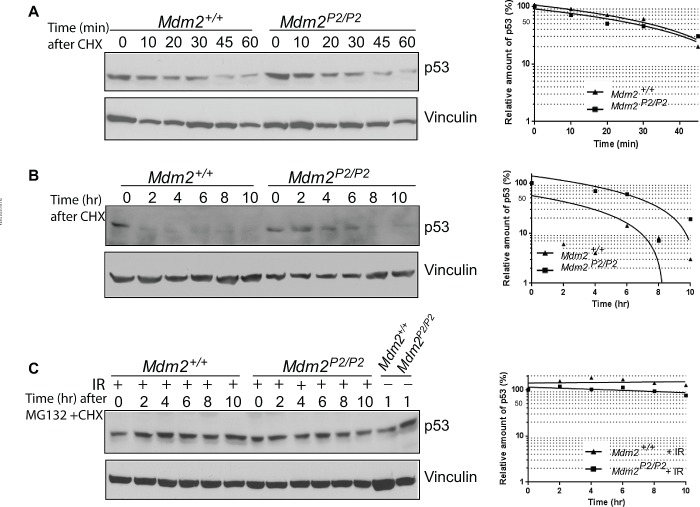
Feedback loop is dispensable for p53 stability (A) Western blot analysis for p53 degradation kinetics in *Mdm2^+/+^* and *Mdm2^P2/P2^* MEFs at different time points. (B) Western blot analysis for p53 degradation kinetics in 6 Gy IR treated *Mdm2^+/+^* and *Mdm2^P2/P2^* MEFs at different time points. (C) Western blot analysis for p53 degradation kinetics in un-irradiated and irradiated *Mdm2^+/+^* and *Mdm2^P2/P2^* MEFs treated with MG132 at different time points. Graphs depict the quantification of the respective western blots (*left*).

Mdm2 is the most important E3 ubiquitin ligase for p53 [6-8]. Therefore, we tested whether the pattern and kinetics of p53 degradation were altered in the absence of the feedback loop. We treated early passage *Mdm2^+/+^* and *Mdm2^P2/P2^* MEFs with Cycloheximide and harvested them at different time points (Figure 2A). A slight enhancement in p53 stability was noticeable in *Mdm2^P2/P2^* MEFs. Nonetheless, the pattern of p53 degradation in both MEF cell lines was similar. Moreover, the kinetics of p53 degradation was also comparable between both MEF genotypes. Notably, p53 half-life in both MEF lines was limited to less than 30 minutes, consistent with previous studies [21]. These experiments suggest that either basal levels of Mdm2 are sufficient or other proteins are involved in p53 degradation.

Next, we examined whether the pattern and kinetics of p53 degradation after DNA damage were altered in feedback loop deficient MEFs. To this effect, we irradiated *Mdm2^+/+^* and *Mdm2^P2/P2^* MEFs with 6 Gy IR and allowed p53 protein to accumulate for 3 hours. Subsequently, we treated cells with Cycloheximide and harvested them at different time points (Figure 2B). Interestingly, while the stabilized p53 was quickly degraded in *Mdm2^+/+^* MEFs, it remained stable for a much longer duration in *Mdm2^P2/P2^* MEFs. The prolonged stability of p53 in *Mdm2^P2/P2^* MEFs implies that stress induced Mdm2 is involved in p53 degradation.

To test this hypothesis, we repeated the above experiments in the presence of MG132, a proteosome inhibitor (Figure 2C). Addition of MG132 completely inhibited p53 degradation in un-irradiated and irradiated *Mdm2^+/+^* and *Mdm2^P2/P2^* MEFs for the duration of the experiment. This confirms that other E3 ligases, in addition to Mdm2, are involved in p53 degradation.

### Role of stress-induced Mdm2 levels in Mdm4 degradation

**Figure 3:**
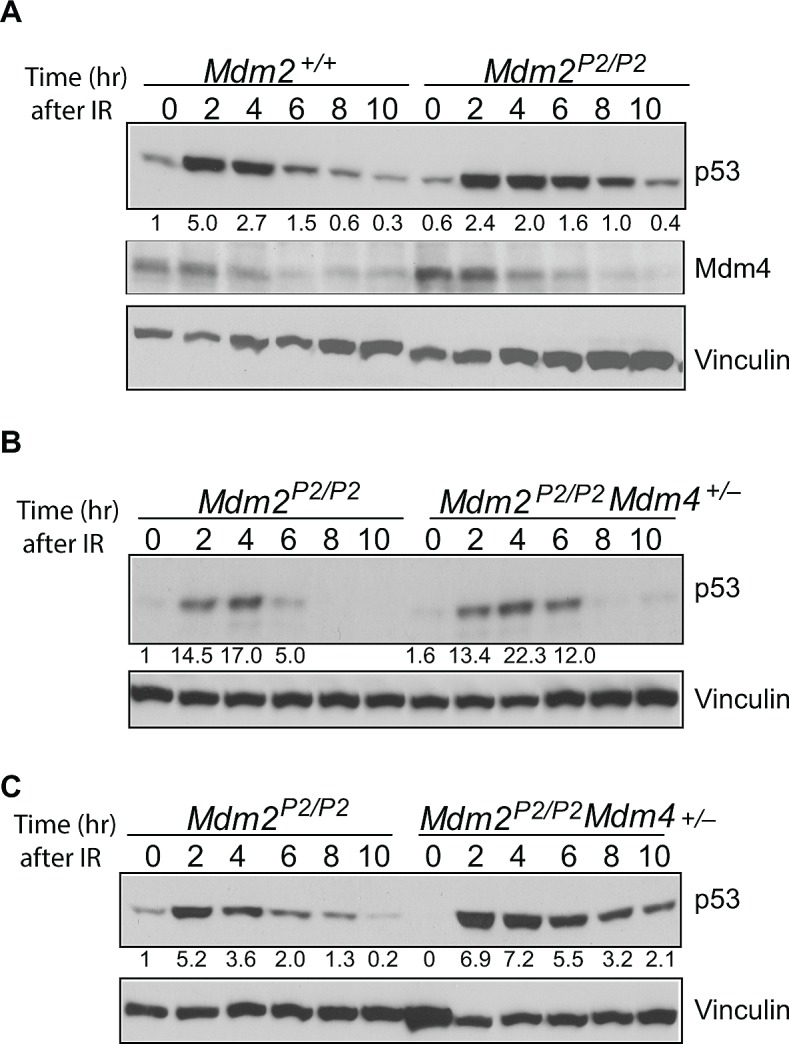
Mdm4 cooperates with Mdm2 in p53 degradation (A) Western blot for p53 and Mdm4 degradation pattern in thymi of irradiated *Mdm2^+/+^* and *Mdm2^P2/P2^* mice at different time points. (B) Western blot analysis for p53 degradation kinetics in spleens of *Mdm2^P2/P2^* and *Mdm2^P2/P2^Mdm4^+/−^* mice at different time points. (C) Western blot analysis for p53 degradation kinetics in thymi of *Mdm2^P2/P2^* and *Mdm2^P2/P2^Mdm4^+/−^* mice at different time points. Numbers at the bottom denote p53 fold induction normalized to vinculin controls and relative to untreated *Mdm2^+/+^* controls.

Mdm4, a structural homolog of Mdm2, is another essential inhibitor of p53 [22]. Although Mdm4 lacks E3 ubiquitin ligase activity, similar to Mdm2, it can bind to p53 and inhibit its transcriptional activity [22]. This interaction is critical for development as confirmed by genetic experiments wherein the embryo lethal *Mdm4*-null phenotype is rescued on a *p53*-null background [5, 23]. Mdm4 is also targeted by Mdm2 E3-ubiquitin ligase activity [21]. RING domain mediated heterodimerization between Mdm2 and Mdm4 is a critical step for this degradation [21, 24]. To investigate whether p53 induced Mdm2 plays a role in Mdm4 degradation, we examined the degradation profiles of p53 and Mdm4 in *Mdm2^+/+^* and *Mdm2^P2/P2^* mouse thymi after IR (Figure 3A). As previously noted in irradiated spleens [14] and MEFs [above], p53 degradation was slightly impeded in *Mdm2^P2/P2^* mouse thymi after IR. However, the degradation pattern of Mdm4 was identical in the two genotypes. These data suggest that basal levels of Mdm2 expressed from the P1-promoter are sufficient to degrade Mdm4.

### Role of Mdm4 in p53 degradation

While Mdm4 is involved in inhibiting p53 activity during development and under stress conditions, its role in degradation of stress-induced p53 is not clear, especially since Mdm4 lacks an inherent E3 ubiquitin ligase activity. One possibility suggests that the role of Mdm4 in p53 degradation is masked by Mdm2 which is the dominant homolog and also a transcriptional target of p53. Since in our model p53-mediated induction of Mdm2 (and hence increases in its level) is blocked, we next examined the role of Mdm4 in stress induced p53 degradation. We asked whether Mdm4 contributes towards Mdm2-mediated p53 degradation. To that end, we crossed *Mdm2^P2/P2^* mice with *Mdm4^+/−^* mice and generated *Mdm2^P2/P2^Mdm4^+/−^* mice. Notably, these mice were born at normal Mendelian ratios with no overt phenotypes. Next, we compared p53 degradation profiles in spleens of *Mdm2^P2/P2^* and *Mdm2^P2/P2^Mdm4^+/−^* after exposure to 6 Gy IR. Importantly, the degradation pattern of p53 in both mouse genotypes remained similar (Figure 3B, 3C). Nonetheless, a slight enhancement in stabilization and delay in degradation of p53 was noticeable in spleens and thymi of *Mdm2^P2/P2^Mdm4^+/−^* mice. This suggests that Mdm4 likely contributes towards p53 degradation through Mdm2 in mouse tissues.

### Mdm4 deficiency promotes p53 activity in *Mdm2^P2/P2^* background

**Figure 4:**
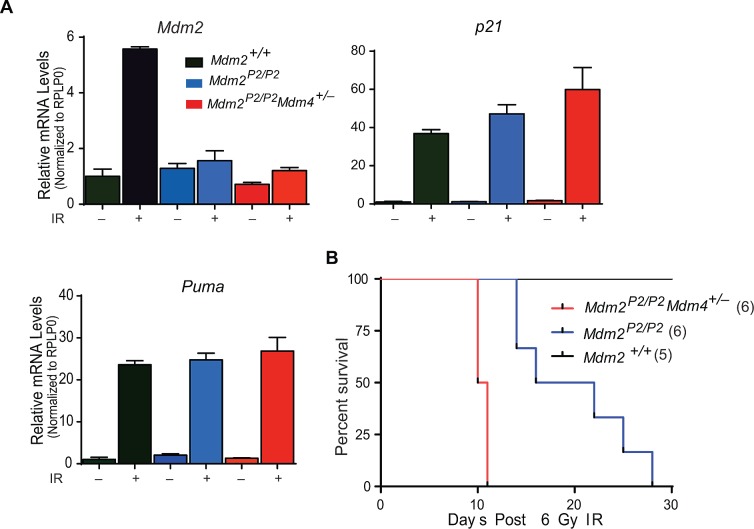
Mdm4 regulates p53 activity (A) RT-qPCR analysis for p53 targets in spleens of *Mdm2^+/+^*, *Mdm2^P2/P2^* and *Mdm2^P2/P2^Mdm4^+/−^* mice after IR (n=3, ±SEM). (B) Kaplan-Meier survival curve of *Mdm2^+/+^*, *Mdm2^P2/P2^* and *Mdm2^P2/P2^Mdm4^+/−^* mice after 6 Gy IR.

We have reported that p53 activity is typically enhanced in *Mdm2^P2/P2^* mice in comparison to wild type mice after DNA damage [14]. Thymus and spleen show enhanced activation of p53 targets after IR. As Mdm4 is also an inhibitor of p53 activity, we next compared p53 transcriptional activity in spleens from *Mdm2^P2/P2^* and *Mdm2^P2/P2^Mdm4^+/−^* mice. As expected, RT-qPCR analysis showed that p53 activity was, but not significantly, enhanced in these mice as compared to *Mdm2^P2/P2^* mice (Figure 4A).

Enhanced p53 activity imparts radiosensitivity in *Mdm2^P2/P2^* mice [14]. While all wild type mice irradiated with 6 Gy IR survive, >80% *Mdm2^P2/P2^* mice exposed to 6 Gy IR die due to bone marrow (BM) failure [14]. To examine whether enhanced p53 activity in *Mdm2^P2/P2^Mdm4^+/−^* mice exacerbates the radiosensitive phenotype, we monitored survival of irradiated mice (Figure 4B). Notably, irradiated *Mdm2^P2/P2^Mdm4^+/−^* mice died much earlier than the *Mdm2^P2/P2^* mice. Early death in response to the low dose radiation suggested hematopoietic failure in these mice. Altogether, these experiments suggest that Mdm4 cooperates with Mdm2 in inhibiting p53 activity as reduction in *Mdm4* gene dosage further enhanced radiosensitivity of *Mdm2^P2/P2^* mice.

### Feedback loop in ageing

The role of p53 in ageing is controversial. Some mouse models with enhanced p53 activity are associated with ageing phenotypes as measured by increased genomic aberrations and decline in stem cell numbers and function [25, 26]. However, other mouse models with increased p53 activity either due to a hypomorphic *Mdm2* allele or due to the expression of an extra copy of the *p53* gene do not exhibit ageing phenotypes [27, 28]. We previously tested whether reduced levels of stress-induced Mdm2 in feedback-defective *Mdm2^P2/P2^* mice also influenced stem cell number and function by quantifying hematopoietic stem cell numbers in wild type and *Mdm2^P2/P2^* mice by flow cytometry [14]. Hematopoietic stem cell numbers (Lin-Sca+Kit+; LSK) in unirradiated bone marrow were similar in both genotypes [14]. Next, to evaluate the effect on lifespan, we monitored a cohort of *Mdm2^P2/P2^* and *Mdm2^+/+^* mice for 800 days [14]. Again, both groups of mice exhibited normal survival and reproduction profiles. No obvious signs of ageing such as lordokyphosis etc. were observed in either genotype. This further confirms that the p53-Mdm2 feedback loop is dispensable for survival and its absence is not detrimental for normal lifespan and functions.

We also investigated whether exposure to a minor genotoxic insult could alter the survival profile of *Mdm2^P2/P2^* and *Mdm2^+/+^* mice. To that end we exposed a cohort of *Mdm2^P2/P2^* and *Mdm2^+/+^* mice (n=10) with 3 Gy IR and monitored them for survival [14]. Again, no difference in survival was evident in either mouse cohort. This suggests that even in the absence of the p53-Mdm2 feedback loop, small increases in p53 activity are well tolerated and do not alter long term stem cell functionality. We have now performed pathology on this cohort and found that 30% (3 out of 10) of the irradiated *Mdm2^P2/P2^* and *Mdm2^+/+^* mice also developed lymphomas. These data concur with a previous report that showed that pathological radiation response is irrelevant for suppression of lymphoma development later in life [29]. Together, these data indicate that enhanced acute p53 activity generated in the absence of the feedback loop does not protect against tumorigenesis.

## DISCUSSION

This study characterizes the p53-Mdm2 feedback loop *in vivo*. Since its discovery in the early nineties, the p53-Mdm2 feedback was considered the main mechanism by which p53 autoregulates its activity and levels to baseline following stress exposure. This was deemed essential for homeostasis and development. Nonetheless, *p53*-null and homozygous transcriptionally-compromised p53 mutant mice are viable [30-32]. This in itself suggests that p53-Mdm2 feedback regulation is dispensable for development. Since the transactivation function of p53 is also compromised in null/mutant mice, we designed novel experiments to test feedback loop functionality. The viability of the *Mdm2^P2/P2^* mouse which has wild type p53 and only lacks p53-mediated Mdm2 induction directly shows that the feedback loop does not play an important role during development. In contrast, mouse models with further reduced Mdm2 levels show increased p53 activity and developmental defects [27]. Therefore, p53 needs to be tightly regulated for normal growth and development. The viability of homozygous *Mdm2^P2/P2^* mice and the lack of any phenotypic aberrations suggest that basal Mdm2 levels from the P1-promoter are sufficient for regulating p53 functions during development.

We also investigated the role of the feedback loop in regulating p53 levels after different types of stress exposures. p53 stabilization and degradation patterns in both *Mdm2^+/+^* and *Mdm2^P2/P2^* mouse tissues were indistinguishable. In addition, changes in the kinetics of p53 degradation in *Mdm2^+/+^* and *Mdm2^P2/P2^* MEFs in the presence or absence of DNA damage were quite modest. Addition of MG132 inhibited p53 degradation indicating that additional E3-ligases are likely involved in p53 degradation. The possibility exists that Mdm2 is restricted to monoubiquitinating p53 while subsequent polyubiquitination and degradation is carried out by other proteins. Conversely, other unidentified protein(s) could be responsible for the degradation of p53 after DNA damage [20]. The *Mdm2^P2/P2^* model provides an excellent system to test these hypotheses.

Another important question addressed in this study is whether stress induced Mdm2 is required for Mdm4 degradation. The degradation pattern of Mdm4 was not altered in *Mdm2^+/+^* and *Mdm2^P2/P2^* genotypes. This suggests that basal Mdm2 levels are sufficient to regulate Mdm4 degradation. To test the role of Mdm4 in stress-induced p53 degradation, we compared the degradation pattern of p53 in *Mdm2^P2/P2^* and *Mdm2^P2/P2^Mdm4^+/−^* spleens. A modest delay in p53 degradation in *Mdm2^P2/P2^Mdm4^+/−^* spleens and thymi was observed. This implicates Mdm4 in p53 degradation. Possibly stabilization of Mdm2 by Mdm4 promotes p53 degradation. Additionally, *Mdm2^P2/P2^Mdm4^+/−^* also exhibit enhanced p53 activity as noted by increase in expression of p53 target genes in the spleen. Survival of these mice after 6 Gy IR was also shortened. These data suggest that Mdm4 cooperates with Mdm2 in regulating p53.

Feedback autoregulation is essential for regulating p53 activity specifically in the hematopoietic system after DNA damage. In its absence, mice are radiosensitized and die due to bone marrow annihilation [14]. However, the lack of a feedback loop does not impact lifespan. Even in the absence of this loop small increases in p53 activity are well tolerated and do not alter stem cell functionality [14]. Moreover, increase in acute p53 activity does not promote ageing phenotypes or confer protection against tumorigenesis.

## MATERIALS AND METHODS

MEF culture and Cycloheximide studies: MEFs were generated and maintained as previously described. Early passage cells (P2-P3) were used for all experiments. Cycloheximide was added at 20ug/ml and cells were harvested at different time points. Protein lysate was prepared in NP-40 lysis buffer containing protease inhibitor cocktail. 100 μg of protein was resolved on 8% SDS-PAGE gel, transferred onto nitrocellulose membrane and bloated with either an, anti-p53 antibody (CM5, Vector biolabs), anti-Mdm4 antibody (MX82, Sigma) or anti-Vinculin antibody (V9131, Sigma). p53 expression was quantitated using Image J software (NIH).

Mouse radiation studies: All animal studies were conducted in compliance with IACUC approved protocols. Mice were radiated at 6 Gy IR and survival curve plotted using graphpad software. For p53 stability experiments, tissues were harvested at different time points, lysed in NP-40 buffer and analyzed by western blotting as described above.

## FUTURE PERSPECTIVES

### Role of p53-Mdm2 feedback loop in other stem cell types

In this and published studies, we have extensively examined the role of the p53-Mdm2 feedback loop in hematopoietic stem cells after DNA damage. Hematopoietic stem cells are essential for maintaining a normal pool of blood cells and sustaining hematopoiesis throughout life. *Mdm2^P2/P2^* mice are extremely sensitive to irradiation and succumb to hematopoietic failure. A simple bone marrow transplant experiment with unirradiated wild type BM cells rescues the *Mdm2^P2/P2^* mouse radiolethality [14]. It will be interesting to test whether stem cells in other tissues are also similarly sensitized in the absence of the feedback loop. More specifically, neural stem cells or spermatogonial stem cells which have been previously shown to be radiosensitive in a p53 dependent manner can be examined for this purpose [33, 34].

In addition to hematopoietic syndrome, gastrointestinal failure is another fatality associated with radiation exposure [35]. Gastrointestinal failure occurs due to degeneration of the intestinal villi and crypts in response to high doses of IR. *p53*-null mice are hypersensitive to GI failure while mice with an extra copy of p53 are protected [35, 36]. However, the role of acute p53 activity in GI protection has not been rigorously examined. It will be interesting to investigate the role of p53-Mdm2 feedback loop and acute p53 activation in GI protection.

### p53-Mdm2 feedback loop in evolution

Evolution of p53 and Mdm2 has been traced back to about 1.5 million years in the placozoans [37]. It is believed that Mdm2 functions primarily as the p53 regulator in all animal species. On the basis of our study, it is easy to comprehend that Mdm2 might be required to inhibit p53 activity in stem cells after DNA damage. This is essential to maintain its genomic integrity function. Unfortunately, p53 responsive *Mdm2* promoters have not been described in all the species. It will be interesting to trace the evolution of the dual *Mdm2* promoters and the feedback loop. Also it is worth noting that Mdm2 is not found in D. *melanogaster* and C. *elegans* while p53 function are essentially the same in both species [37]. While the reasons for this anomaly are not clear, they warrant a thorough investigation. Perhaps, introducing a codon justified Mdm2 in these animals can provide insights into the role of feedback loop here.

### E3-ubiquitin ligases for degradation of stress induced p53

While many E3-ubiquiting ligases have been identified for p53 degradation only Mdm2 has been verified by *in vivo* studies. Here we examined the role of Mdm2 in degradation of p53 after DNA damage. The slightly delayed but eventual degradation of p53 in *Mdm2^P2/P2^* mice suggests that Mdm2 may not be sufficient by itself to degrade stress-induced p53. As addition of MG132, a proteosome inhibitor drug, inhibits p53 degradation in MEFs after IR, this suggests that other E3- ligases might be involved. It is also possible that Mdm2 is primarily involved in monoubiquitinating p53 and the mark is recognized by other unidentified E3 ligases that promote polyubiquitination and complete degradation of p53 by the proteosome machinery. *Mdm2^P2/P2^* mice/cells with a defective feedback loop can be used to test these hypotheses.

### Inhibition of p53-Mdm2 interaction as a therapeutic strategy

Our results show that hematopoietic system is extremely sensitive to p53 activity variations. HSC share many characteristics with leukemia stem cells [38]. Therefore, transient activation of p53 by inhibiting the p53-Mdm2 feedback loop in conjunction with DNA damage could be an effective therapeutic strategy for sensitizing stem cells in these malignancies. This could be a safer alternative to high-dose radio/chemotherapy regimens which have bystander effects. In fact, similar ideas are currently being tested in the clinic [39].

## CONCLUSIONS

Our studies suggest that the feedback loop is dispensable for inhibiting p53 activity during development but is critical for regulating p53 activity in HSCs after DNA damage. In contrast to the existing paradigm, the feedback loop is *not* as important for regulating p53 stability. This suggests that other yet unknown E3 ligases might play a more important or compensatory role in p53 stability. The *Mdm2^P2/P2^* mouse presents a unique opportunity to identify these E3 ligases and to test other hypotheses involving p53 regulation by Mdm2 and Mdm4.
